# Integrated Transcriptomic and Proteomic Analysis Associated with Knockdown and Overexpression Studies Revealed ECHDC1 as a Regulator of Intramuscular Fat Deposition in Cattle

**DOI:** 10.3390/ani15243558

**Published:** 2025-12-11

**Authors:** Ruiying He, Li Liu, Xianya Kong, Nanfei Wang, Jianbing Tan, Zhangqing Wu, Linsen Zan, Wucai Yang

**Affiliations:** 1 College of Animal Science and Technology, Northwest A&F University, Yangling 712100, China; 2 Shenzhen Research Institute, Northwest A&F University, Shenzhen 518000, China

**Keywords:** cattle, IMF content, transcriptome, proteome, *ECHDC1*

## Abstract

Intramuscular fat (IMF) content is a critical determinant of beef quality and is regulated by various factors. However, the molecular mechanisms underlying IMF deposition in cattle remain poorly understood, particularly with regard to multi-omics integrated analyses. In this study, an integrative analysis of transcriptomic and proteomic profiles was performed on intramuscular fat tissue of Jiaxian Red cattle with different marbling grades to identify key genes and pathways involved in intramuscular fat deposition in beef cattle. Integrated analysis showed that 4532 genes were co-expressed at both mRNA and protein level, among which 21 genes exhibited significant differential expression at mRNA and protein level. These genes showed significant enrichment in lipid biosynthesis and metabolic processes. ECHDC1 was selected for in-depth functional studies on bovine intramuscular preadipocyte adipogenesis as a candidate gene via RNA interference and overexpression techniques. The results indicated that knockdown of ECHDC1 inhibited the adipogenesis of bovine intramuscular preadipocytes and significantly upregulated the expression of HSL. Conversely, overexpression of ECHDC1 promoted the adipogenesis of bovine intramuscular preadipocytes, decreased the expression of HSL and increased the expression of FABP4. In summary, these results suggested that ECHDC1 promoted intramuscular fat deposition by attenuating lipolysis rate by downregulating the expression of the lipolytic gene HSL, providing a potential molecular target for enhancing IMF content in beef cattle.

## Simple Summary

Intramuscular fat (IMF) content is a critical determinant of beef flavor, tenderness, and juiciness. It is regulated by many factors, including nutrition, non-coding RNA, and genetic factors, among which, genetic factors play a key role. Therefore, identifying key genes influencing intramuscular fat deposition in beef cattle and clarifying their regulatory mechanisms are of great significance for improving beef quality. In this study, integrated transcriptomic and proteomic analyses were performed on longissimus dorsi muscle of Jiaxian Red cattle with different marbling grades and identified 21 differentially expressed genes (DEGs). These DEGs were enriched in lipid synthesis and metabolic processes. Gain-of-function and loss-of-function experiments confirmed that the differentially expressed gene ECHDC1 promoted adipogenic differentiation of bovine preadipocytes by downregulating the expression of the lipolysis-related gene HSL. These findings provided important theoretical support for increasing intramuscular fat content and optimizing breeding strategies in beef cattle.

## 1. Introduction

With the rapid economic and social development, the global demand for high-quality beef has increased significantly. Intramuscular fat content is a key indicator for evaluating beef quality, and its accumulation significantly impacts beef grading [1]. Preadipocytes derived from mesenchymal stem cells in the bone marrow serve as the fundamental building blocks of adipose tissue [2]. Under the combined influence of cytokines, signaling pathways, hormones, and inductive factors (like insulin), mesenchymal stem cells (MSCs) can be directed to differentiate into preadipocytes [3,4]. Numerous functional genes, including *PPARγ*, *C*/*EBPα*, *FABP4*, and *SREBP-1*, as well as key signaling pathways such as PPAR, mTOR, MAPK, and Wnt, have been identified as critical regulators in preadipocyte differentiation [5,6]. For instance, CEBPδ and CEBPβ can induce PPARγ expression, which in turn activates CEBPα and downstream adipogenesis-related genes, thereby promoting intramuscular fat deposition [7]. Furthermore, studies have confirmed that fatty acid metabolism also plays an important role in intramuscular fat deposition. Fatty acid metabolism-related genes exhibit differential expression in the longissimus dorsi muscle of different cattle breeds, suggesting their potential involvement in regulating bovine intramuscular fat deposition [8]. Ethylmalonyl-CoA decarboxylase 1 (ECHDC1) has been shown to regulate the synthesis of branched-chain fatty acids in adipocytes [9], which may affect adipogenesis and beef quality. However, the specific role of ECHDC1 in bovine intramuscular fat deposition remains unreported. Although existing studies have identified several genes and pathways regulating bovine intramuscular fat deposition, the critical regulatory genes involved in this process still require systematic exploration, and the associated molecular mechanisms need to be further elucidated. Multi-omics technologies have emerged as a powerful tool in recent years, enabling the integration of diverse omics data and surpassing the limitations of single-omics approaches [10]. Multi-omics studies are conducive to unraveling the genetic basis of complex traits in cattle, including the molecular mechanisms underlying important economic traits such as growth, reproduction, health, and environmental adaptability [8,11,12]. In the field of meat quality improvement, multi-omics studies systematically decipher the genetic regulatory networks, key metabolic pathways, and phenotypic formation mechanisms of intramuscular fat (IMF) deposition by integrating multi-dimensional data from genomics, transcriptomics, proteomics, and metabolomics, thereby offering core molecular evidence for precise beef cattle breeding. Specifically, genomics can identify genetic markers associated with fat traits, providing critical clues for the mining of candidate functional genes [13]. Transcriptomics focuses on comparative analysis of animal samples with varying IMF contents, aiming to screen key differentially expressed genes (DEGs) or non-coding RNAs that regulate adipogenesis [14]. However, the correlation between mRNA and protein abundance is relatively low [15], so relying solely on transcriptomic data is insufficient to fully reflect the actual expression and function of genes. Proteomics can address this limitation by analyzing the expression characteristics, post-translational modifications (PTMs), and functional states of proteins, providing direct evidence for screening core functional proteins related to fat traits. Furthermore, metabolomics can identify volatile flavor compounds and key metabolic pathways affecting meat flavor [16], offering targeted nutritional regulation strategies for meat quality improvement. The integrated application of the aforementioned multi-omics technologies enables a more comprehensive and systematic interpretation of the formation mechanisms of meat quality traits. For example, Deng et al. demonstrated that intramuscular fat content in yaks varies significantly across different dietary regimens, and they elucidated the mechanism by which lipid–gene interactions regulate intramuscular fat deposition using integrated transcriptome and metabolome analyses [17]. Additionally, Yan et al. conducted a combined transcriptomic and proteomic analysis to identify differentially expressed genes and proteins in the longissimus dorsi muscle of adult castrated bulls from Xinjiang Brown cattle and Kazakh cattle [18]. Nevertheless, existing studies mostly focus on mining functional genes and conducting bioinformatics analyses across different breeds or developmental stages [19,20,21]. There is a lack of multi-omics research on populations within the same breed under consistent management and at the same developmental stage, making it difficult to rule out interference from genetic background. Moreover, most studies merely identify differential molecules without functional validation, resulting in numerous discoveries failing to be translated into reliable targets [22,23]. Therefore, integrating transcriptomics and proteomics to investigate the key regulatory mechanisms of intramuscular fat deposition is particularly significant for improving beef quality and intramuscular fat content.

In this study, we selected Jiaxian Red cattle with distinct marbling grades as the research model. Marbling beef refers to high-quality beef with intramuscular fat evenly deposited to form marble-like textures. As an elite indigenous beef cattle breed in China, Jiaxian Red cattle possesses desirable traits such as rapid growth, robust roughage tolerance, and high stress resistance. Notably, this breed exhibits outstanding meat quality with prominent intramuscular fat deposition capacity, distinct marbling features and a mature and comprehensive marbling grading system, making it an ideal experimental material for deciphering the molecular mechanisms underlying intramuscular fat deposition in beef cattle. In the present study, we conducted an integrative analysis of transcriptomic and proteomic profiles in intramuscular fat tissues with different marbling grades to identify key genes and pathways involved in intramuscular fat deposition in beef cattle. Additionally, the role of ECHDC1 in this process was validated. The findings discussed herein reveal, from multiple dimensions, the dynamic changes in genes associated with intramuscular fat deposition at both mRNA and protein levels within the same breed. These results lay a theoretical foundation for elucidating the molecular mechanisms underlying intramuscular fat deposition in cattle and supplement the research gap in this field. Meanwhile, this study provides reliable molecular targets for the precise regulation of intramuscular fat deposition, which is conducive to accelerating the breeding process for fat traits in beef cattle.

## 2. Materials and Methods

### 2.1. Animals, Samples and Ethical Approval

The intramuscular fat tissue used for transcriptome and proteome sequencing were obtained from Jiaxian Red cattle with different marbling grades (A2 and A4; graded using the beef marbling scorecard), all raised under the same feeding conditions (Pingdingshan Ruibao Red Beef Industry Co., Ltd., Pingdingshan, Henan, China). The tissue samples including heart, lung, kidney, pericardial fat, perirenal fat, subcutaneous fat, mesenteric fat and longissimus dorsi muscle (M. longissimus thoracis et lumborum) were harvested from three-year-old Jiaxian Red Beef. The fresh tissue samples were stored at −80 °C for subsequent study. All operations in this research strictly abide by the ordinances of the Administration of Laboratory Animals (Ministry of Science and Technology, China, revised 2004).

### 2.2. RNA Extraction, Sequencing, and Transcriptome Analysis

Total RNA was extracted from intramuscular fat tissue samples from Jiaxian Red cattle of A2 and A4 grades, with 3 biological replicates per grade. RNA purity and quality was assessed via a NanoDrop 2000 spectrophotometer (Thermo Fisher Scientific, Waltham, MA, USA) and an Agilent 4200 Bioanalyzer (Agilent Technologies, Santa Clara, CA, USA), and RNA integrity was determined through agarose gel electrophoresis. High-quality RNA was randomly fragmented into short segments ranging from 200 to 500 nucleotides (nt) via high-temperature treatment at 94 °C for 5 min. These fragments were used to synthesize the first and second strands of cDNA. The resulting double-stranded cDNA underwent end repair, A-tailing, and ligation of sequencing adapters. Subsequently, the adapter-ligated products were purified using VAHTS DNA Clean Beads to select target fragments. The purified cDNA was then subjected to PCR amplification with 10–17 cycles for product enrichment and cDNA library construction. Finally, sequencing analysis was performed using the Illumina HiSeq™ 4000 platform.

Filtering and processing were performed on the completed raw sequencing data. Fastp software (v0.18.0, https://github.com/OpenGene/fastp, accessed on 5 January 2024) was used for quality control to remove low-quality sequences and adapter sequences. On this basis, HISAT2 software (v2.1.0, https://daehwankimlab.github.io/hisat2/, accessed on 12 January 2024) was employed to align the filtered data with the reference genome, so as to obtain the localization information of the sequences on the reference genome. StringTie software (v1.3.4, https://ccb.jhu.edu/software/stringtie/, accessed on 16 January 2024) was utilized to identify the genes annotated in the reference genome as well as novel genes. The expression levels of all genes in each sample were quantified using the TPM (Transcripts Per Million) metric for intuitive presentation of expression abundance. Differential expression analysis was performed using the DESeq2 software (v1.38.3, https://bioconductor.org/packages/release/bioc/html/DESeq2.html, accessed on 19 January 2024) based on the read counts of transcripts in each sample, with a false discovery rate (FDR) threshold of <0.05 and an absolute log_2_ fold change (|log_2_FC|) of >1. These criteria were applied to identify differentially expressed genes (DEGs) among intramuscular fat tissues from marbling beef with different grades. Subsequently, GO (http://www.geneontology.org/, accessed on 21 January 2024) and KEGG (http://www.kegg.jp/, accessed on 21 January 2024) pathway analysis were conducted on the selected DEGs to elucidate their biological functions and associated metabolic pathways.

### 2.3. Protein Extraction, Sequencing, and Proteomics Analysis

Filtering and processing were performed on the completed raw sequencing data. Fastp software (v0.18.0, https://github.com/OpenGene/fastp, accessed on 7 January 2024) was used for quality control to remove low-quality sequences and adapter sequences. On this basis, HISAT2 software (v2.1.0, https://daehwankimlab.github.io/hisat2/, accessed on 13 January 2024) was employed to align the filtered data with the reference genome, so as to obtain the localization information of the sequences on the reference genome. StringTie software (v1.3.4, https://ccb.jhu.edu/software/stringtie/, accessed on 15 January 2024) was utilized to identify the genes annotated in the reference genome as well as novel genes. Subsequently, the database construction was completed using the Pulsar software (v1.8, https://www.biognosys.com/software/spectronaut/, accessed on 19 January 2024) [24]. For the qualitative analysis of proteins, the criteria for protein identification were set as Precursor FDR ≤ 0.01 and Protein FDR ≤ 0.01. For the quantitative analysis of proteins, the peak areas of peptide segments with FDR less than 1.0% were selected. Missing values in protein quantitative data were imputed with 0 or mean values. The rules were as follows: if the number of samples with missing values in a group exceeds ½, the missing values were imputed with 0; if not, they were imputed with the group mean. Subsequent data analysis was performed using the total table of expression levels after imputation. A t-test was conducted on protein abundance, defining proteins with |log_1.2_FC| > 1 and *p*-value < 0.05 as differentially expressed proteins (DEPs) [25,26]. Finally, Gene Ontology (GO) annotation and Kyoto Encyclopedia of Genes and Genomes (KEGG) enrichment analysis were performed on the identified DEPs.

### 2.4. Isolation and Induced Differentiation of Bovine Intramuscular Preadipocytes

Intramuscular preadipocytes were isolated from the longissimus dorsi muscle of newborn Qinchuan cattle provided by the National Beef Cattle Improvement Center according to previously published methods in our laboratory [27]. The isolated preadipocytes were cultured in the complete medium containing 90% DMEM/F12 medium (Hyclone, Logan, UT, USA), 10% fetal bovine serum (PAN-Biotech, Aidenbach, Germany), 1% penicillin and streptomycin (Hyclone, Logan, UT, USA). The complete medium was refreshed every two days. The cells were inoculated in 6-well plates (NEST, Wuxi, China) and cultured in the incubator (Thermo Fisher Scientific, Waltham, MA, USA) at 37 °C and 5% CO_2_. When the cell density reached 100%, cultured the cells were cultured with adipogenic differentiation induction medium (complete medium supplemented with 5 μg/mL insulin (Solarbio, Beijing, China), 1 μM dexamethasone (Sigma-Aldrich, St. Louis, MO, USA), and 1 μM rosiglitazone (Sigma-Aldrich, St. Louis, MO, USA)). The medium was refreshed every two days during cell culture.

### 2.5. Cell Transfection

siRNAs were designed based on the coding sequence (CDS) region of ECHDC1 in the sequencing data. Meanwhile, the CDS region was digested with HindIII and EcoRI restriction enzymes and cloned into the pcDNA3.1(+) vector to construct the ECHDC1 overexpression plasmid. siRNA-ECHDC1, OE-ECHDC1, and their respective negative controls (si-NC and OE-NC, purchased from Sangon Biotech, Shanghai, China; sequence information is provided in Table 1) were transfected into intramuscular preadipocytes using Lipofectamine™ 3000 reagent (Thermo Fisher Scientific, Waltham, MA, USA), with three replicates per group (*n* = 3).

### 2.6. Total RNA Extraction and RT-qPCR

Total RNA was extracted from IMF using TRIzol reagent (Takara, Dalian, China). The RNA quality was assessed using NanoDrop 2000, and samples with an A260/A280 optical density ratio between 1.8 and 2.0 were considered acceptable. Total RNA was reverse-transcribed into cDNA using a reverse transcription kit (Takara, Dalian, China). RT-qPCR was performed using a real-time quantitative PCR kit (Takara, Dalian, China) to analyze gene relative expression (primer sequences are listed in Table 2. Primers were designed using Primer Premier 5.0 (Premier Biosoft, Palo Alto, CA, USA) and synthesized by TsingKe Biotechnology (Xian, China). The 18S gene was used as a reference gene to normalize the mRNA expression of target genes, and the 2^−ΔΔCt^ method was applied to calculate relative expression levels.

### 2.7. Oil Red O Staining and Triglyceride (TG) Content Assay

After the cell treatment was completed, the culture medium was discarded, and cells were washed three times with PBS (Solarbio, Beijing, China). Cells were fixed with 4% paraformaldehyde (Solarbio, Beijing, China) at room temperature for 30 min, and then washed three times with PBS. Lipid droplets in the cells were stained with Oil Red O working solution (Sigma, St. Louis, MO, USA) in the dark for 30 min. Subsequently, images were captured using an inverted microscope. Total triglycerides were extracted using the tissue-cell triglyceride enzymatic assay kit (APPLYGEN, Beijing, China), and protein quantification was performed with the BCA protein quantification kit (APPLYGEN, Beijing, China). The cellular triglyceride was normalized to corresponding protein concentration.

### 2.8. Western Blot

Total protein was extracted with WB/IP lysis buffer (Beyotime, Shanghai, China) containing 1 mM phenylmethylsulfonyl fluoride (PMSF, Solarbio, Beijing, China) on ice for 30 min. Protein concentration was determined by the BCA protein quantification kit (APPLYGEN, Beijing, China). Proteins were then mixed with loading buffer (Biosharp, Hefei, China) and denatured at 100 °C for 10 minutes. Total protein was separated using 12% SDS-PAGE gels (Solarbio, Beijing, China), and then transferred onto polyvinylidene fluoride (PVDF) membranes (Millipore, Burlington, MA, USA). The protein was blocked with BLOT-QuickBlocker™ (Beyotime, Shanghai, China) for half an hour. Primary antibodies were incubated overnight at 4 °C, followed by two hours of secondary antibody incubation. Finally, chemiluminescent horseradish peroxidase substrate (Millipore, Bedford, MA, USA) was added. Images were acquired via the Bio-Rad Molecular Imager (Bio-Rad, Hercules, CA, USA). Antibody information is provided in Table S1. β-actin was used as an internal reference protein.

### 2.9. Statistical Analysis

Data analysis and visualization were performed using GraphPad Prism (v9.0, https://www.graphpad.com/, accessed on 20 May 2024). Results were presented as mean ± standard error of the mean (SEM). Differences between two groups were analyzed using an unpaired t-test. Differences between multiple groups were analyzed using one-way ANOVA, followed by Tukey’s test for post hoc analysis. Statistical significance was defined as *p* < 0.05, while *p* > 0.05 was considered not significant (* and ** denote *p* < 0.05 and *p* < 0.01, respectively; ns denotes *p* > 0.05).

## 3. Results

### 3.1. Transcriptomic Analysis Based on Differentially Expressed Genes

**Figure 1:**
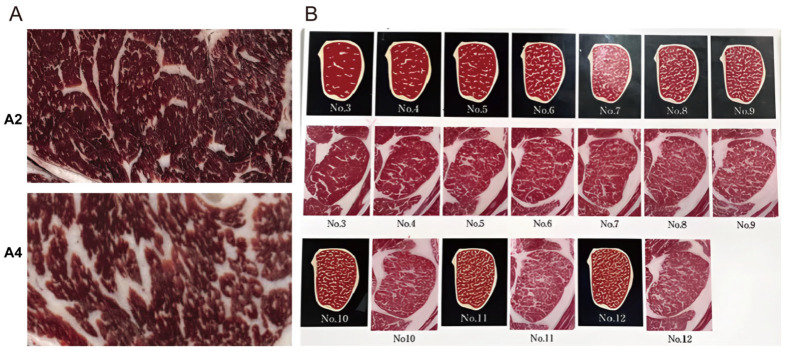
Comparison of beef with different intramuscular fat content. (**A**) Photographs of beef at grades A2 and A4. (**B**). Beef Marbling Standard (BMS) comparison chart. Beef grades are determined by both yield grade (grades A/B/C, with grade A having the highest meat yield) and meat quality grade. The meat quality grade is evaluated based on four core indicators: marbling degree, meat color, fat color, and meat firmness. Grades A1–A5 correspond to BMS Grade 1, Grade 2, Grades 3–4, Grades 5–7, and Grades 8–12, respectively, with meat quality gradually improving with increasing grade.

**Figure 2:**
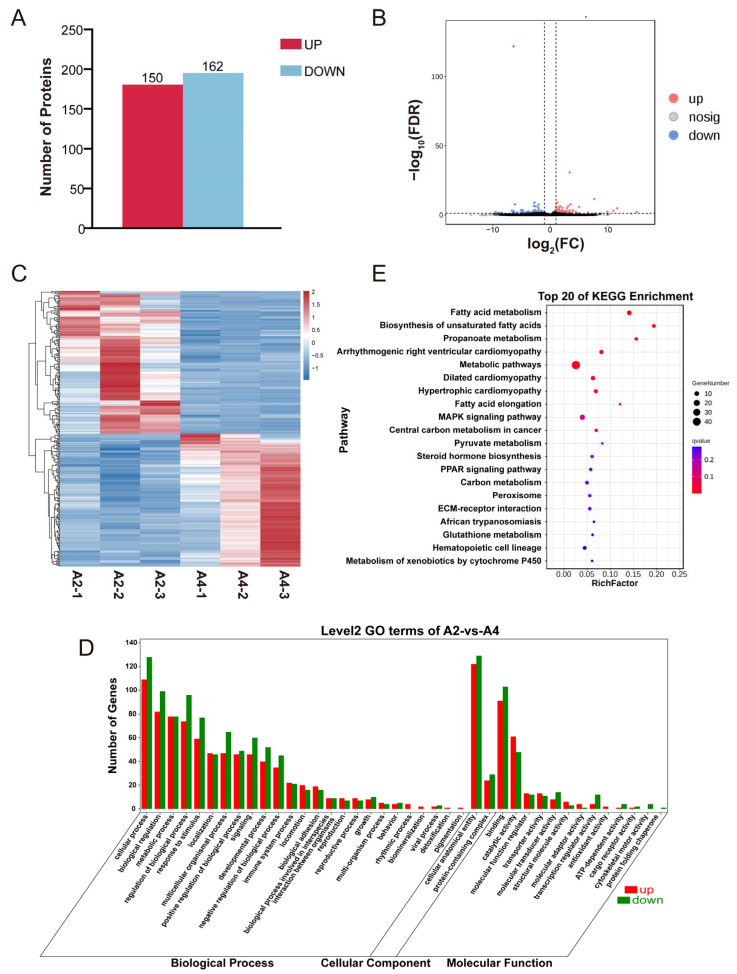
Differential expression and enrichment analysis of mRNA. (**A**) Differential gene bar chart. (**B**) Differential gene volcano plot. Vertical dotted lines on the horizontal axis denote FC thresholds of 2 and 0.5. Horizontal dotted line on the vertical axis indicates an FDR threshold of 0.05. (**C**) Differential gene clustering heatmap. (**D**) Histogram of differentially expressed mRNAs functional GO enrichment. (**E**) Differentially expressed mRNAs KEGG bubble chart.

Based on the significance level of differences, a total of 312 differentially expressed mRNAs were identified between the A2 and A4 groups, of which 150 were significantly upregulated and 162 were significantly downregulated (Figure 1 and Figure 2A,B). Hierarchical clustering analysis of the differentially expressed genes (DEGs) revealed a trend of clustering within the same group (Figure 2C). GO enrichment analysis of the DEGs indicated that the aforementioned DEGs were significantly enriched in 82 subcategories: including 17 cellular components, 3 molecular functions, and 62 biological processes (Figure 2D). In the cellular component category, cell components such as fatty acid elongase complexes were significantly enriched; in terms of molecular function, 11% of the DEGs showed significant enrichment in oxidoreductase activity, 7% of the DEGs were significantly enriched in actin binding function, and 2.33% of the DEGs were enriched in oxidoreductase activity acting on the CH-CH group of donors; and regarding biological processes, DEGs were primarily associated with small molecule metabolism, lipid metabolism, oxoacid metabolism, monocarboxylic acid metabolism, and lipid biosynthesis. KEGG enrichment analysis showed that DEGs were significantly enriched in lipid metabolism-related signaling pathways, including PPAR, MAPK, fatty acid metabolism, and biosynthesis of unsaturated fatty acids (Figure 2E). These results provide important information for exploring the key regulatory mechanisms of snowflake beef meat quality traits of marbling beef of marbling beef at the transcriptional level.

### 3.2. Proteomics Analysis Based on Differential Proteins

**Figure 3:**
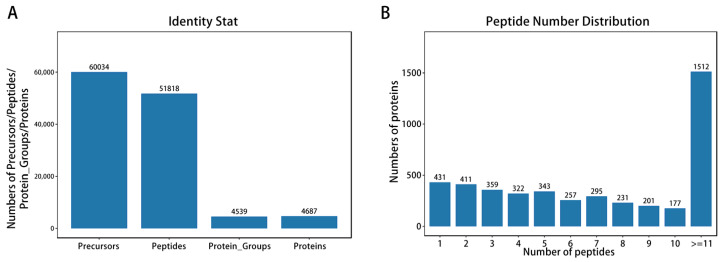
Quantitative and qualitative analysis of proteins. (**A**) Protein identification results. (**B**) The number of identified peptide segments.

To further explore the mechanism of intramuscular fat deposition, we conducted LC-MS/MS analysis on the proteome of A2 and A4 grade marbled beef with Data-independent Acquisition (DIA) technology. A total of 60,034 spectra were identified, corresponding to 51,818 peptide segments, ultimately determining 4687 proteins, the majority of which contained 11 or more peptide segments (Figure 3A,B).

**Figure 4:**
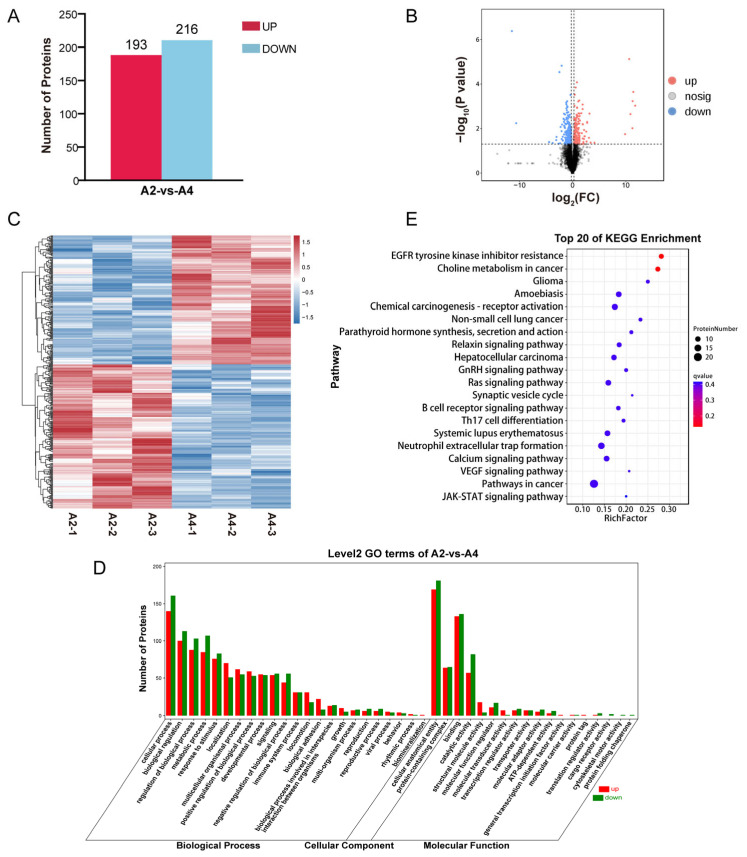
Differential expression and enrichment analysis of proteins. (**A**) Differential protein bar chart. (**B**) Differential protein volcano plot. Vertical dotted lines on the horizontal axis denote FC thresholds of 2 and 0.5. Horizontal dotted line on the vertical axis indicates an FDR threshold of 0.05. (**C**) Differential protein clustering heatmap. (**D**) The bar chart of functional GO enrichment for differentially expressed proteins. (**E**) Functional enrichment KEGG analysis of genes differentially expressed proteins.

Taking |log_1.2_FC| > 1 and *p*-value < 0.05 as the criteria, a total of 409 differentially expressed proteins (DEPs) were identified in the intramuscular fat tissues of two grades of marbling beef. Compared to the A2 group, there were 193 DEPs significantly upregulated and 216 DEPs significantly downregulated in the A4 group (Figure 4A,B). Moreover, the differential clustering heatmap visually illustrated the variation in DEPs abundance across different grades of marbling beef (Figure 4C). Subsequently, we mapped DEPs to the GO database for functional annotation and enrichment analysis. The results indicated that the enriched cellular component mainly included trans-Golgi network transport vesicle membranes and mitochondrial proton-transporting ATP synthase complex. In terms of molecular function, DEPs showed a strong association with lipase activator activity; as for biological progresses, DEPs mainly involved responses to interferon-alpha and responses of cells to extracellular stimuli (Figure 4D). KEGG enrichment analysis revealed that DEPs were significantly enriched in disease occurrence, signal transduction, metabolic processes and cellular functions (Figure 4E).

### 3.3. Analysis of the Association Between Transcriptome and Proteome

**Figure 5:**
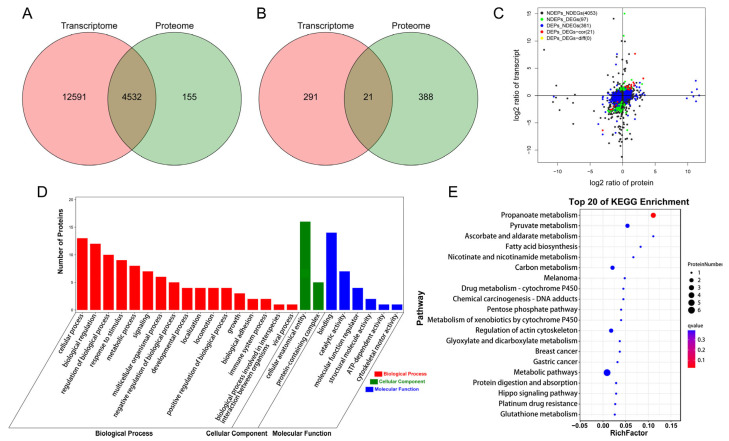
Analysis of the association between Transcriptome and Proteome. (**A**) Venn diagram of all genes and proteins detected. (**B**) Venn diagram of DEGs and DEPs. (**C**) Transcriptomics and proteomics association analysis quadrant diagram. (**D**) GO enrichment bar chart of DEGs/DEPs. (**E**) KEGG bubble chart of DEGs/DEPs.

To gain a comprehensive understanding of the expression patterns of differentially expressed genes, we conducted a combined analysis of the transcriptome and proteome of intramuscular adipocytes. Venn diagrams illustrate the overlap between transcripts and proteins (Figure 5A,B), with 4532 genes expressed at both transcript and protein levels, among which 21 genes showed significant expression differences at both levels. A quadrants analysis was performed to assess the association between transcriptome and proteome data. As shown in Figure 5C, the 21 co-expressed DEGs/DEPs exhibited a consistent trend in expression changes, with 15 DEGs/DEPs (*SNCG*, *ECHDC1*, *FGF1*, *CLEC11A*, *PHYHIPL*, *RIDA*, *ACSS2*, *ACACA*, *QPRT*, *CDH2*, *COL3A1*, *H2AC11*, *GSTA3*, *RGN*, *TMSB15A*) being upregulated and 6 DEGs/DEPs (*FIBIN*, *PPP1R12B*, *PDK4*, *IFITM1*, *CFH*, *MYH6*) being downregulated. GO and KEGG enrichment analyses were performed on the DEGs/DEPs with consistent expression trends. The results from the GO database revealed that these genes were enriched in processes such as growth factor activity, lipid biosynthetic process, muscle tissue morphogenesis, and acetyl-CoA metabolic process (Figure 5D). The KEGG enrichment analysis indicated that the co-expressed DEGs/DEPs were significantly enriched in the propanoate metabolism pathway (Figure 5E), with *ACSS2*, *ACACA*, and *ECHDC1* participating in this pathway. Additionally, co-expressed DEGs/DEPs were also enriched in pathways such as pyruvate metabolism, fatty acid biosynthesis, and the pentose phosphate pathway.

### 3.4. Temporal and Tissue ECHDC1 Relative mRNA Levels

**Figure 6:**
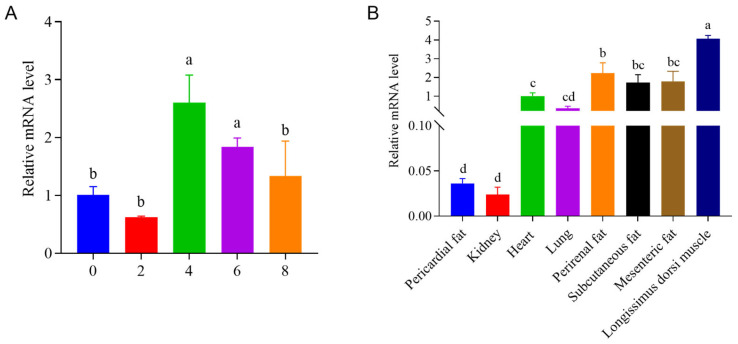
Temporal and tissue relative mRNA level of ECHDC1. (**A**) Relative mRNA levels of ECHDC1 on days 0, 2, 4, 6, and 8 of bovine intramuscular preadipocyte differentiation. (**B**) Relative mRNA level of ECHDC1 in various tissues of cattle. *n* = 3, identical lowercase letters indicate no significant difference between groups, and different lowercase letters indicate significant differences.

This study investigated the relative mRNA levels of the key candidate gene ECHDC1 by RT-qPCR, finding that its expression peaked on the fourth day after the differentiation of bovine intramuscular preadipocytes (Figure 6A) and exhibited highest relative mRNA levels in the longissimus dorsi muscle tissue among multiple tissues (Figure 6B). This mRNA expression pattern indicates that ECHDC1 may play a critical role in intramuscular preadipocyte differentiation and intramuscular fat deposition.

### 3.5. Attenuation of ECHDC1 Inhibited the Adipogenesis of Intramuscular Preadipocytes

**Figure 7:**
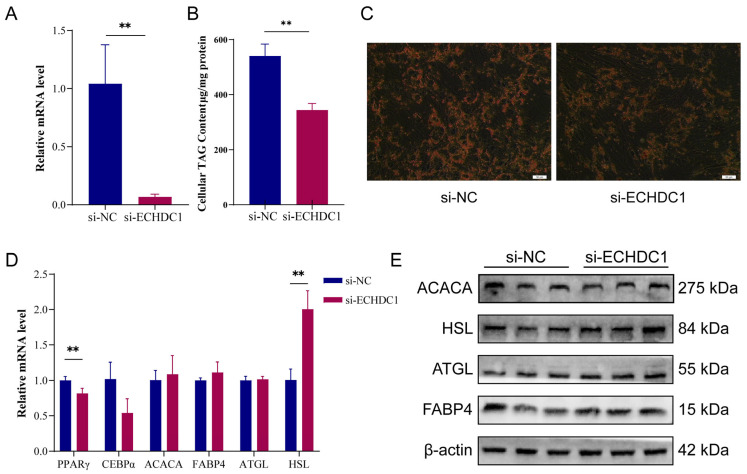
Attenuation of ECHDC1 inhibited lipid synthesis. (**A**) The expression level of ECHDC1 after 48 h when ECHDC1 was knocked down. (**B**) Total triglyceride content in intramuscular preadipocytes treated with si-ECHDC1. (**C**) The lipid droplets in intramuscular preadipocytes treated with si-ECHDC1. (**D**) The mRNA expression level of genes related to lipid metabolism in intramuscular preadipocytes treated with si-ECHDC1. (**E**) The protein expression level of genes related to lipid metabolism in intramuscular preadipocytes treated with si-ECHDC1. Full uncropped Western blot scans are provided in Supplementary Figure S1A. *n* = 3, ** represents *p* < 0.01.

To investigate the role of ECHDC1 in lipid accumulation, bovine intramuscular preadipocytes were transfected with siRNA targeting *ECHDC1* or with a scramble si-RNA (si-NC). After 48 h of transfection, the mRNA level of ECHDC1 in the si-ECHDC1 group was significantly downregulated by 90% compared to the si-NC group (*p* < 0.01) (Figure 7A). The Oil red O staining results showed that si-ECHDC1 inhibited lipid droplets accumulation in intramuscular preadipocytes (Figure 7C). The TG assay results demonstrated that the triglyceride content was lower in the si-ECHDC1 group compared with the si-NC group (Figure 7B). These results indicate that knockdown of ECHDC1 inhibited the adipogenesis of intramuscular preadipocytes.

To further elucidate the role of ECHDC1 in lipid metabolism, we evaluated the expression of genes related to lipogenesis and lipolysis after ECHDC1 knockdown. RT-qPCR and Western blot analyses revealed that knockdown of ECHDC1 significantly downregulated the mRNA level of the key lipogenic gene *PPARγ* (*p* < 0.01) and significantly upregulated the mRNA and protein level of the lipolytic gene *HSL* (*p* < 0.01) (Figure 7D,E).

### 3.6. Overexpression of ECHDC1 Promoted the Adipogenesis of Intramuscular Preadipocytes

**Figure 8:**
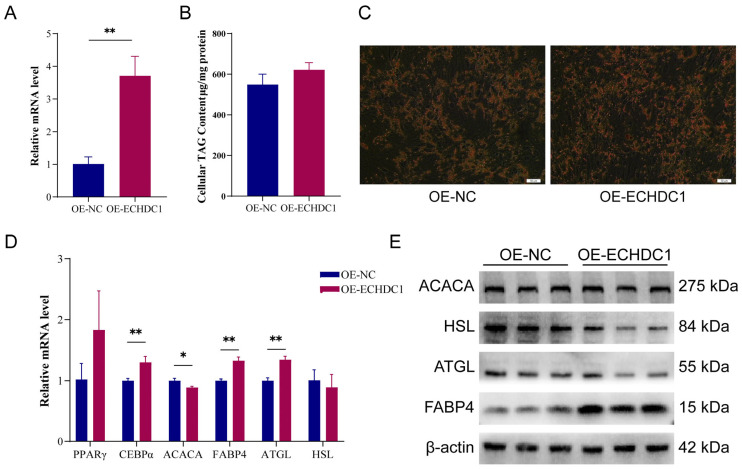
The overexpression of ECHDC1 promotes lipid synthesis. (**A**) The expression level of ECHDC1 in intramuscular preadipocytes treated with OE-ECHDC1 after 48 h. (**B**) Total triglyceride content in intramuscular preadipocytes treated with OE-ECHDC1. (**C**) The lipid droplets in intramuscular preadipocytes treated with OE-ECHDC1. (**D**) The mRNA expression level of genes related to lipid metabolism in intramuscular preadipocytes treated with OE-ECHDC1. (**E**) The protein expression level of genes related to lipid metabolism in intramuscular preadipocytes treated with OE-ECHDC1. *n* = 3, * represents *p* < 0.05, ** represents *p* < 0.01. Full uncropped Western blot scans are provided in Supplementary Figure S1B.

The overexpressed plasmid of ECHDC1 (OE-ECHDC1) was designed and transfected into bovine intramuscular preadipocytes. The results showed that the expression level of ECHDC1 in the OE-ECHDC1 group was significantly upregulated compared to overexpression negative control (OE-NC) (*p* < 0.01) (Figure 8A). The results of Oil Red O staining and TG assay found that the number of lipid droplets (Figure 8C) and TAG level (Figure 8B) in OE-ECHDC1 group exhibited an upward trend compared to the OE-NC group. The RT-qPCR and Western Blot analyses showed that overexpression of ECHDC1 significantly upregulated the mRNA and protein level of lipogenesis-related gene *FABP4* (Figure 8D). Meanwhile, the RT-qPCR result showed the overexpression of ECHDC1 significantly elevated the *ATGL* expression (Figure 8D), while there was no significant change in the ATGL protein level (Figure 8E). Although ECHDC1 overexpression did not significantly alter *HSL* mRNA levels (Figure 8D), it markedly reduced HSL protein levels (Figure 8E). Together, these results suggest that ECHDC1 overexpression may promote lipid accumulation by upregulating the lipid-synthesis–related gene *FABP4* and suppressing the lipolysis-related gene *HSL*.

## 4. Discussion

The intramuscular fat content is an important criterion for assessing the grade of beef, as it influences flavor, juiciness, tenderness, and overall taste, which in turn significantly affects consumer preferences [28,29]. Therefore, in-depth exploration of the key regulatory mechanisms underlying the deposition of intramuscular fat is particularly important for increasing the intramuscular fat content and meat quality of beef. With the continuous advancement of high-throughput sequencing technology, the combined application of transcriptomics and proteomics has become a crucial approach for elucidating the molecular mechanisms of complex traits in animals [30,31]. This study aims to analyze the transcriptome and proteome of intramuscular fat tissue from beef with different marbling grades, in order to elucidate differential gene expression and reveal its potential mechanisms in the formation of meat quality traits.

In this study, the 312 DEGs identified were enriched in pathways related to lipid synthesis, such as fatty acid metabolism, biosynthesis of unsaturated fatty acids, PPAR signaling pathway, and MAPK signaling pathway. The PPAR signaling pathway comprises PPARα, PPARβ/δ, and PPARγ. Among them, PPARγ is a key regulatory factor in adipocyte differentiation, lipid metabolism, and glucose metabolism [32,33]. The DEPs were primarily enriched in diseases, signal transduction, metabolic processes, and cellular functions, among which choline metabolism, calcium signaling, and JAK-STAT signaling pathway are directly related to lipid metabolism [34,35]. For example, the calcium cycle regulates beige fat thermogenesis through an enhanced ATP-dependent calcium cycling mechanism mediated by sarcoplasmic reticulum/endo-plasmic reticulum calcium ATPase 2b and Ryanodine receptor 2 [36]; JAK2-STAT3 pathway is activated in the early process of adipogenesis [12] and may promote adipocyte differentiation by regulating the transcription of C/EBPβ [37]. The integrated analysis of the DEGs and DEPs revealed that 21 genes exhibited significant differences at both the transcriptional and protein levels, with consistent trends in their expression providing a reliable foundation for subsequent functional analyses.

Unlike the mitochondria in brown adipose tissue, the mitochondria in white adipose tissue are specialized for nutrient storage [38]. Mitochondrial dynamics in white adipose tissue provide energy for the de novo synthesis of fatty acids and glycerol-3-phosphate, thereby influencing the synthesis of triglycerides [39]. This study conducted GO and KEGG enrichment analyses on DEGs/DEPs, finding that these genes were enriched in metabolic pathways such as pyruvate metabolism, fatty acid biosynthesis, and the pentose phosphate pathway. The mitochondrial pyruvate carrier (MPC) regulates cellular nutrient allocation by controlling the entry of pyruvate into the mitochondria, thereby affecting pyruvate metabolism [37]. Under aerobic conditions, pyruvate enters the mitochondria through the MPC and undergoes decarboxylation under the catalysis of the pyruvate dehydrogenase complex to form acetyl-CoA, which then enters the tricarboxylic acid cycle [36,40]. Research has found that interference with MPC can suppress the ability to synthesize lipids and glycerol-3-phosphate, leading to a reduction in triglyceride accumulation [36,41]. In addition, pyruvate can also synthesize acetyl-CoA through the mitochondrial-cytoplasmic MPC–ATP–citrate lyase pathway, initiating de novo synthesis of cytoplasmic fatty acids [42]. The aforementioned studies indicate that pyruvate metabolism plays a significant role in fat deposition. This study found through combined analysis that ECHDC1 was differentially expressed in intramuscular fat tissues of beef with varying grades, and previous studies found that interference with this gene inhibited the number of intracellular lipid droplets [43]. However, existing studies have not clearly explored the regulatory role of the ECHDC1 gene in the adipogenesis of bovine intramuscular adipocytes, and the potential interaction between ECHDC1 and other lipid metabolism-related genes remains to be further clarified. Based on the above research gaps, this study hypothesizes that the ECHDC1 gene may play a key regulatory role in bovine intramuscular fat deposition and intends to initially investigate its molecular regulatory mechanisms.

ECHDC1 is a cytoplasmic enzyme that catalyzes the decarboxylation of ethyl or methyl malonyl-CoA [44], thereby playing a role in fatty acid synthesis and restricting the synthesis of abnormal fatty acids, such as ethyl or methyl-branched fatty acids [9,45]. This study analyzed the spatiotemporal specificity of ECHDC1 and found that it may be involved in the early stages of preadipocyte differentiation. Its high expression in the longissimus dorsi muscle tissue further suggests that it may play a role in intramuscular fat deposition. To clarify the impact of ECHDC1 on the differentiation of bovine intramuscular preadipocytes, we conducted interference and overexpression experiments. The results showed that si-ECHDC1 inhibited the adipogenesis of intramuscular preadipocytes and significantly upregulated the mRNA and protein expression of HSL. HSL is a key enzyme that catalyzes the hydrolysis of triglycerides into free fatty acids and glycerol, and its expression is positively regulated by signaling pathways such as the cAMP-dependent PKA pathway and AMPK [46]. Previous studies have demonstrated that ECHDC1 functions in fatty acid β-oxidation [43], suggesting that it may inhibit HSL expression via the AMPK pathway by enhancing intracellular fatty acid β-oxidation activity and increasing cellular energy levels. Furthermore, experiments on ECHDC1 overexpression confirmed that ECHDC1 could significantly reduce HSL protein levels, implying that ECHDC1 may promote HSL degradation by activating the ubiquitin-proteasome system of HSL. Taken together, we speculate that ECHDC1 may participate in fat deposition via regulation of the expression of the lipolysis-related gene *HSL*, while the specific regulatory mechanism remains to be further investigated. Although this study confirmed that ECHDC1 promotes adipogenesis in intramuscular preadipocytes via in vitro functional experiments, its regulatory effect in vitro may not fully reflect its real function in the in vivo physiological environment. Future studies should construct ECHDC1 gene-edited cattle models to further validate its regulatory role in intramuscular fat deposition in vivo, thereby providing more reliable theoretical support for beef cattle quality improvement. These findings provide a potential molecular target for enhancing IMF content in beef cattle. In the future, sustained validation of key candidate genes identified through multi-omics is required to facilitate their translation into precision breeding targets. Furthermore, multi-omics technologies offer substantial scope for further exploration in the genetic improvement of fat traits in beef cattle, and there is an urgent need for in-depth dissection of the “gene-transcription-translation-metabolism” cascade regulatory network.

## 5. Conclusions

In conclusion, 21 genes exhibited significant differential expression at both transcriptional and protein levels and were significantly enriched in lipid biosynthesis and metabolic processes in intramuscular fat tissues with different marbling grades. Our findings in in vitro-cultured preadipocytes suggest that ECHDC1 facilitates intramuscular fat deposition by attenuating lipolysis rate through downregulating the expression of the lipolytic gene *HSL*. The differentially expressed genes identified in this study provide important clues for identifying key candidate genes associated with fat traits. Combining these candidate genes with biological breeding technologies such as gene editing holds promise for the directional regulation of intramuscular fat deposition in beef cattle, thereby further enhancing the market value of beef.

## Figures and Tables

**Table 1:** Sequence of siRNA for ECHDC1 bovine.

Name	Sense Sequence (5′-3′)	Antisense Sequence (5′-3′)
si-NC	UUCUCCGAACGUGUCACGUTT	ACGUGACACGUUCGGAGAATT
si-ECHDC1	GCAUGUGAUUUCAGGUUAATT	UUAACCUGAAAUCACAUGCTT

**Table 2:** Primers for RT-qPCR.

Genes	Primer Sequence	Annealing Temperature	NCBI RefSeq Accession
18S	F: CCTGCGGCTTAATTTGACTC	61 °C	NR_036642.1
	R: AACTAAGAACGGCCATGCAC		
ECHDC1	F: ATCATCGGCGGTAGACAAGC	61 °C	/
	R: TGGTCCACCCCAAACTGTTC		
PPARγ	F: TGAAGAGCCTTCCAACTCCC	61 °C	NM_181024.2
	R: GTCCTCCGGAAGAAACCCTTG		
C/EBPα	F: ATCTGCGAACACGAGACG	61 °C	NM_176784.2
	R: CCAGGAACTCGTCGTTGAA		
HSL	F: GATGAGAGGGTAATTGCCG	61 °C	NM_001080220.1
	R: GGATGGCAGGTGTGAACT		
ATGL	F: TGCTGATTGCTATGAGTGTGCC	61 °C	NM_001046005.2
	R: CCTCTTTGGAGTTGAAGTGGGT		
FABP4	F: GCTGCACTTCTTTCTCACCTTG	61 °C	NM_174314.2
	R: ACCACACCCCCATTCAAACT		
ACACA	F: CTCCAACCTCAACCACTACGG	61 °C	NM_174224.2
	R: GGGGAATCACAGAAGCAGCC		

“/” indicates that the ECHDC1 primers were designed based on its coding sequence obtained from RNA-seq.

## Data Availability

The datasets used and/or analyzed during the current study are available from the corresponding author upon reasonable request.

## Supplementary Materials

The following supporting information can be downloaded at: https://doi.org/10.5281/zenodo.17242096. Figure S1: Full Scans of the Western Blots; Table S1: Antibody information.

## Abbreviations

The following abbreviations are used in this manuscript:

IMF	intramuscular fat
DEG	differentially expressed gene
DEP	differentially expressed protein
siRNA	small interfering RNA
CDS	Coding DNA Sequence
ATP	Adenosine Triphosphate
MPC	Mitochondrial Pyruvate Carrier
TAG/TG	Triacylglycerol
DMEM/F12	Dulbecco’s Modified Eagle Medium/F12 Nutrient Mixture
MSC	mesenchymal stem cell
ECHDC1	Enoyl-CoA Hydratase Domain-Containing Protein 1
HSL	hormone-sensitive lipase
FABP4	fatty acid binding protein 4
PPARγ	peroxisome proliferator-activated receptor γ
C/EBPα	CCAAT/enhancer binding protein
ACACA	Acetyl-CoA carboxylase Alpha
ATGL	adipose triglyceride lipase
SREBP-1	Sterol Regulatory Element-Binding Protein-1
SNCG	Synuclein Gamma
ECHDC1	Enoyl-CoA Hydratase Domain Containing 1
FGF1	Fibroblast Growth Factor 1
CLEC11A	C-Type Lectin Domain Family 11 Member A
PHYHIPL	Phytanoyl-CoA Hydroxylase Interacting Protein-Like
RIDA	RAD51 Inhibitor Domain-Containing Protein
ACSS2	Acyl-CoA Synthetase Short-Chain Family Member 2
QPRT	Quinolinate Phosphoribosyltransferase
CDH2	Cadherin 2 (N-Cadherin)
COL3A1	Collagen Type III Alpha 1 Chain
H2AC11	H2A Clustered Histone 11
GSTA3	Glutathione S-Transferase Alpha 3
RGN	Regucalcin
TMSB15A	Thymosin Beta 15A
FIBIN	Fibulin 1
PPP1R12B	Protein Phosphatase 1 Regulatory Subunit 12B
PDK4	Pyruvate Dehydrogenase Kinase 4
IFITM1	Interferon-Induced Transmembrane Protein 1
CFH	Complement Factor H
MYH6	Myosin Heavy Chain 6 (Alpha-Myosin)
mTOR	mammalian target of rapamycin
MAPK	Mitogen-Activated Protein Kinase
Wnt	Wingless-Type MMTV Integration Site Family
JAK-STAT	Janus Kinase-Signal Transducer and Activator of Transcription
TPM	Transcripts Per Million
FDR	false discovery rate
FC	fold change
GO	Gene Ontology
KEGG	Kyoto Encyclopedia of Genes and Genomes
LC-MS/MS	liquid chromatography-tandem mass spectrometry
DIA	Data-Independent Acquisition
RT-qPCR	Real-time Fluorescence Quantitative PCR
PBS	Phosphate-Buffered Saline
SDS-PAGE	Sodium Dodecyl Sulfate-Polyacrylamide Gel Electrophoresis
BCA	Bicinchoninic Acid Assay
